# NOP58 induction potentiates chemoresistance of colorectal cancer cells through aerobic glycolysis as evidenced by proteomics analysis

**DOI:** 10.3389/fphar.2023.1295422

**Published:** 2023-12-12

**Authors:** Feifei Wang, Bin Yu, Quanyong Yu, Guanglin Wang, Baokun Li, Ganlin Guo, Handong Wang, Hui Shen, Shujin Li, Chunling Ma, Xianxian Jia, Guiying Wang, Bin Cong

**Affiliations:** ^1^ Hebei Key Laboratory of Forensic Medicine, Innovation Center of Forensic Medical Molecular Identification, College of Forensic Medicine, Collaborative Hebei Medical University, Shijiazhuang, Hebei, China; ^2^ The Second Department of Surgery, The Fourth Hospital of Hebei Medical University, Shijiazhuang, Hebei, China; ^3^ Research Unit of Digestive Tract Microecosystem Pharmacology and Toxicology, Chinese Academy of Medical Sciences, Hebei Medical University, Shijiazhuang, Hebei, China; ^4^ China Pharmaceutical University, Nanjing, China; ^5^ Department of Pathogen Biology, Institute of Basic Medicine, Hebei Medical University, Shijiazhuang, China; ^6^ Department of Surgery, The Second Hospital of Hebei Medical University, Shijiazhuang, Hebei, China

**Keywords:** colorectal cancer, chemoresistance, aerobic glycolysis, 5-FU, NOP58

## Abstract

**Introduction:** The majority of individuals diagnosed with advanced colorectal cancer (CRC) will ultimately acquire resistance to 5-FU treatment. An increasing amount of evidence indicates that aerobic glycolysis performs a significant function in the progression and resistance of CRC. Nevertheless, the fundamental mechanisms remain to be fully understood.

**Methods:** Proteomic analysis of 5-FU resistant CRC cells was implemented to identify and determine potential difference expression protein.

**Results:** These proteins may exhibit resistance mechanisms that are potentially linked to the process of aerobic glycolysis. Herein, we found that nucleolar protein 58 (NOP58) has been overexpressed within two 5-FU resistant CRC cells, 116-5FuR and Lovo-5FuR. Meanwhile, the glycolysis rate of drug-resistant cancer cells has increased. NOP58 knockdown decreased glycolysis and enhanced the sensitivity of 116-5FuR and Lovo-5FuR cells to 5FU.

**Conclusion:** The proteomic analysis of chemoresistance identifies a new target involved in the cellular adaption to 5-FU and therefore highlights a possible new therapeutic strategy to overcome this resistance.

## 1 Introduction

Colorectal cancer (CRC) ranks as the third most common prevalent form of cancer globally and it holds the second position in terms of death associated with malignancies (Sung et al., 2021; Heide et al., 2022; Zhuo et al., 2023). Chemotherapy is considered the primary therapeutic approach for individuals with CRC who have had disease progression and metastasis. One often employed strategy involves the administration of 5-fluorouracil (5-FU), either as a standalone agent or as a crucial constituent of systemic chemotherapy, having the purpose of treating CRC (Correale et al., 2004; Rosmarin et al., 2014; Huang et al., 2023; Li et al., 2023). Nevertheless, drug resistance is the greatest challenge for 5-FU treatment. Hence, it is crucial to elucidate the fundamental mechanism responsible for 5-FU resistance.

Cancer cells have enhanced glycolysis as a means to generate ATP, resulting in a transfer in the primary location of energy generation from the mitochondria to the cytosol (DeBerardinis and Chandel, 2016; Kim et al., 2020), which is called the Warburg effect (Kato et al., 2018; Duan et al., 2023). The metabolic changes described herein perform an essential function in facilitating the provision of energy and serving as a source of building blocks, thereby significantly contributing to the evolution of tumors and the development of resistance to chemotherapy (Montal et al., 2015; Shukla et al., 2017; Romani et al., 2022). A rising body of studies suggests that the suppression of glycolysis is a highly effective means of inducing death in multidrug-resistant cells. This finding suggests that directing efforts on glycolysis could serve as a promising and innovative technique for overcoming multidrug resistance (Xu et al., 2005; Li et al., 2022).

Nucleolar protein 58 (NOP58) ribonucleoprotein, performs a crucial function in the maintenance of cellular homeostasis by serving as a critical component for multiple box C/D short nucleolar RNAs (Zhou et al., 2017; Ojha et al., 2020; Abel et al., 2021). Previous studies have provided evidence indicating that NOP58 has the capacity to enhance the stability of family with sequence similarity 83 member A (FAM83A) mRNA, hence facilitating the advancement of tumor growth (He and Yu, 2019), while the suppression of NOP58 was demonstrated to impede the proliferation of cancer cells and reduce their oncogenic properties (Su et al., 2014). Moreover, NOP58 has the ability to directly engage with lncRNA ZFAS1, hence facilitating its functional activation, and this complex ultimately contribute to CRC tumorigenesis (Wu et al., 2020). While the previous research has shown the molecular mechanism of NOP58, its potential involvement in chemoresistance is still unknown.

Hence, the primary purpose of the current research was to characterize the differences between the 5-FU resistant cell lines and wide type on the proteomic level. Here, we showed a metabolic reprogramming mechanism that enables CRC cells to develop resistance to 5-FU treatment. The expression of NOP58 was observed to be significantly increased in 5-FU resistant cell and silencing NOP58 increased the sensitivity to 5-FU through regulation of glycolysis. The current study purpose is to investigate the effect of NOP58 on colon cancer cells that exhibit resistance to 5-FU, thereby offering a novel theoretical framework for potential therapeutic strategies for individuals with 5-FU resistance.

## 2 Materials and methods

### 2.1 5-FU resistance cell model construction

HCT116 and Lovo cell lines underwent a progressive increase in 5-FU concentration, ranging from 1 μg/mL to 10 μg/mL, in a continuous manner. The assessment of resistance to 5-FU was conducted at various doses through the determination of the IC50 utilizing MTS assays. The resistance to 5-FU was operationally defined as a resistance index (RI, IC50 of the 5-FU-R cells/IC50 of the WT cells) > 5. This definition was applied following a 6-month period of 5-FU treatment.

### 2.2 MTS assay

Cells that had undergone pre-treatment under different circumstances were collected and subsequently inoculated within 96-well plates at 4000 cells per well, reaching the final volume of 100 μL per well. Following a 12 h incubation period, 100 μL of the specified concentration of 5-FU was introduced into the wells of 96-well plates. Following a 48 h treatment period, a volume of 13 μL of MTS solution (Sigma; #75-79-6) solution (2 mg/mL) was introduced to all wells and subsequently underwent incubation for a duration of 2 h at 37°C. Using a microplate reader, absorbance was measured at 492 nm.

### 2.3 Mass spectrometry-based proteome profiling

#### 2.3.1 Protein isolation and digestion

The RIPA buffer (Thermo Fisher Scientific, 89901) was introduced to both the 5-FU-R and WT cells in order to facilitate protein extraction. Subsequently, sonication was performed for a total of 6 cycles, with each cycle consisting of 5 s on and 5 s off. The proteins were subsequently subjected to denaturation at 95°C for 2 min. The insoluble fragment was separated from the solution using centrifugation at 12000 *g* for a duration of 10 min. The supernatant was then used in the proteomic investigation. The protein content was determined utilizing BCA kit (Thermo Fisher Scientific, 23227).

The protein digesting process employed the filter-aided sample preparation (FASP) approach. In this study, proteins have been transferred to 10 kDa centrifugal filter tubes (Thermo Fisher Scientific, 88513) and subjected to a series of treatments. Firstly, the disulfide bond was disrupted by incubating the proteins via 50 mM DTT (Sigma-Aldrich, D9163) in 300 μL of UA buffer (8 M urea (Sigma-Aldrich, U0631) in 0.1 M Tris-HCl (Thermo Fisher Scientific, 15506017), pH 8.5) at 37°C for 30 min. Afterwards, the proteins were alkylated by treating them via 50 mM IAA (Sigma-Aldrich, I6125) in 300 μL of UA buffer for a duration of 30 min within the absence of light. Following this, the proteins were washed three times with 300 μL of UA buffer, and then underwent hydration two times using 300 μL of 50 mM NH4HCO3(Sigma-Aldrich, 11213). The aforementioned stages were subjected to centrifugation at 12000 *g* at a temperature of 25°C. The proteins were subjected to enzymatic digestion using trypsin (Thermo Fisher Scientific, 90058) at 1:100 (w/w) in 50 mM NH4HCO3, and the digestion process was carried out at 37°C for 18 h. Following the process of digestion, the elution of peptides was achieved through the utilization of centrifugation. Following that, the peptides underwent purification and extraction utilizing custom-made C18 Tips (Empore, 98060402173) in a solution consisting of 80% ACN and 2% TFA. The peptides underwent lyophilization and were subsequently acidified using a 0.1% FA solution. Using the BCA peptide quantification kit (Thermo Fisher Scientific, 23275), the concentration of the peptide was measured.

#### 2.3.2 Proteomic analysis

In order to perform proteomic analysis, the peptides (1 μg each specimen) were introduced into a nanoflow HPLC Easy-nLC1200 system (Thermo Fisher Scientific), utilizing a 90 min LC gradient at 300 nL/min. 0.1% (v/v) FA was diluted in H2O to prepare Buffer A, while Buffer B was prepared by diluting 0.1% (v/v) FA in 80%ACN. The gradient was established according to the following parameters: 2%–8% B in 1 min; 8%–28% B in 60 min; 28%–37% B in 14 min; 37%–100% B in 5 min; 100% B in 10 min. The Q Exactive HF mass spectrometer (Thermo Fisher Scientific) was utilized for the proteomic analyses. In positive ion mode, the spray voltage was set at 2100 V, while the temperature of the ion transfer tube was kept constant at 320°C. The data-dependent acquisition method was implemented via Xcalibur software, specifically utilizing the profile spectrum data type. The MS1 full scan was configured with 60000 at m/z 200, AGC target 3e6, and maximum IT 20 ms using the orbitrap mass analyzer (350–1500 m/z). This was subsequent by the acquisition of “top 20” MS2 scans by HCD fragmentation, which were performed at 15000 atm/z 200, an AGC target of 1e5, and a maximum IT time of 45 ms. 110,0 m/z was determined to be the initial mass of the MS2 spectrum. The isolation window was established at a value of 1.6 m/z. The experimental conditions included a normalized collision energy (NCE) of 27% and a dynamic exclusion duration of 45 s. Precursors possessing a charge of 1, 8, and >8 was omitted from the MS2 analysis.

### 2.4 Database searching of MS data

The initial processing of data was conducted in Proteome Discoverer 2.2, utilizing an ion-current-based label-free quantification approach. Using Sequest HT with a maximum mass tolerance of 10 ppm for the parent ion and a fragment tolerance of 0.02 Da for tandem mass spectrometry, peptides were identified. The UniProtSwissProt Human canonical database (downloaded on Uniprot, 2022) was utilized to conduct a comprehensive search of all available data. The static modification of cysteines through carbamido methylation was taken into consideration, whereas the potential variable modifications of protein N-termini through acetylation and methionine through oxidation were examined. The application of false discovery rate calculations was employed to conduct multiple testing correction. The false discovery rate threshold for both peptide spectral matches was set at 1%, (which were determined utilizing Percolator), and the peptide group levels. By performing pairwise analysis on individual peptides, the quantification ratios of each peptide were determined and afterwards calculating the average for both peptide groups and protein levels. The determination of significance was thereafter conducted through the utilization of analysis of variance, which was in accordance with the assessment of peptide background at the peptide group and protein levels.

### 2.5 Knockdown of NOP58

GenePharma (Suzhou, China) was the supplier of the small interfering RNA (siRNA) and negative control (NC) targeting NOP58 (the sequence of siRNA in Supplementary Table S1). The transfection procedure was carried out using Lipofectamine 2000 (Invitrogen, Carlsbad, California) according to the manufacturer guidelines.

### 2.6 Total RNA extraction and real-time quantitative PCR (RT-qPCR)

The total RNA extraction was conducted utilizing Trizol reagent (Invitrogen) following the manufacturer’s guidelines. The total RNA that was obtained was quantified using a Nanodrop spectrophotometer, and subsequently converted into cDNA using reverse transcription. The relative quantification of mRNA was conducted using the 2^−ΔΔCt^ approach, with the gene Actin functioning as an internal reference. The sequence of primers is shown in the Supplementary Table S2.

### 2.7 Statistical analysis

The experimental data is reported as the mean ± standard deviation (SD) obtained from three separate and independent studies. The data were subjected to analysis utilizing SPSS 13.0. One-way ANOVA or the Student’s t-test was utilized in the statistical analysis. Statistical significance was established at a *p*-value <0.05.

## 3 Results

### 3.1 Construction and classification of 5-FU-resistant CRC cell lines

**FIGURE 1:**
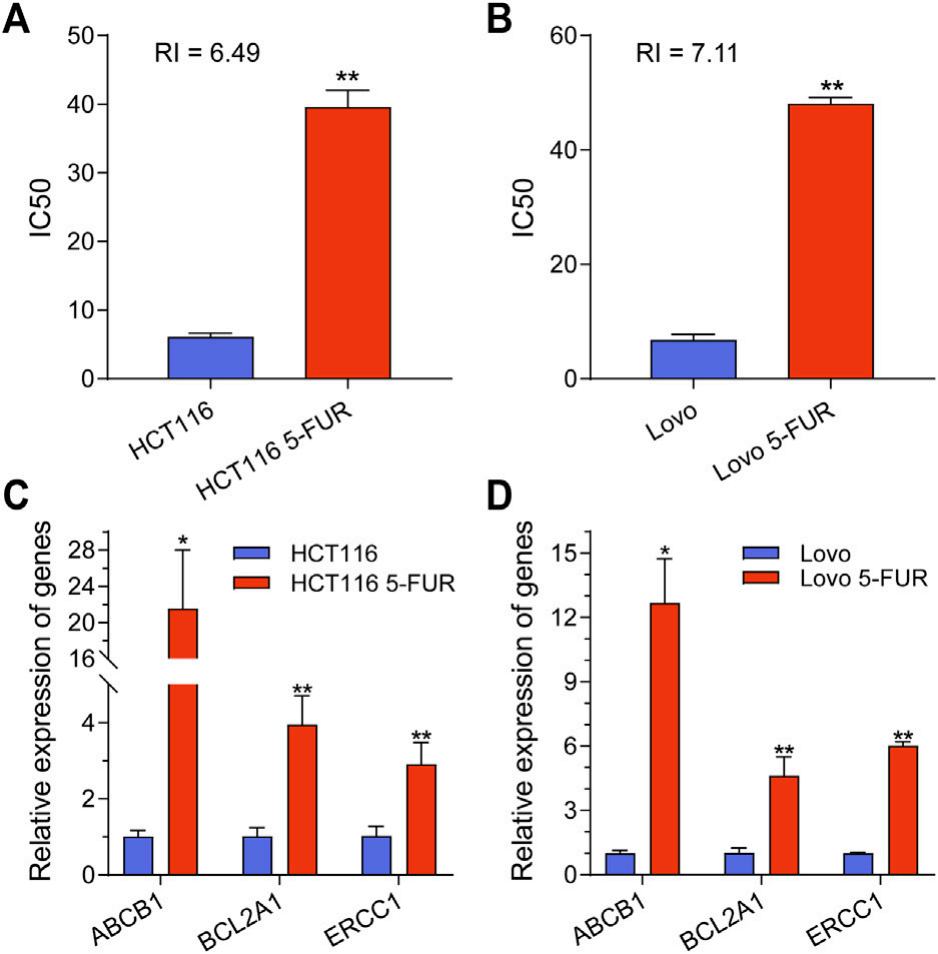
Construction and classification of 5-FU-resistant CRC cell lines. **(A)** IC50 values for parental and 5FU-resistant cells of HCT116 cell. **(B)** IC50 values for parental and 5FU-resistant cells of Lovo cell. **(C)** The mRNA expression level of ABCB1, BCL2A1 and ERCC1 in parental and 5-FU-resistant cells of HCT116. **(D)** The mRNA expression level of ABCB1, BCL2A1 and ERCC1 in parental and 5-FU-resistant cells of Lovo. **p < 0.01, *p < 0.05.

In order to examine the underlying mechanism of 5-FU resistance within CRC, two CRC cell line models (HCT116 and Lovo) with acquired resistance to 5-FU were created. During the course of roughly 8 months, wild-type (WT) cells were exposed to a cultivation method in which the concentration of 5-FU was gradually raised. The half maximum inhibitory concentration (IC50) was determined, revealing that 5-FU resistance (5-FU-R) cells exhibited resistance indexes ranging from 5 to 8 following 5-FU induction. These resistance indexes were assessed subsequent to a 2 weeks period of drug removal (Figures 1A,B). Moreover, drug-resistant associated proteins were detected. The results showed that ABCB1, BCL2A1, and ERCC1 were significantly increased in 5-FU-R CRC cells compared to WT cells (Figures 1C,D). The collective results of this study demonstrate that 5-FU-R CRC cells were successfully established *in vitro*.

### 3.2 Differential genes in 5-FU resistant CRC cells

**FIGURE 2:**
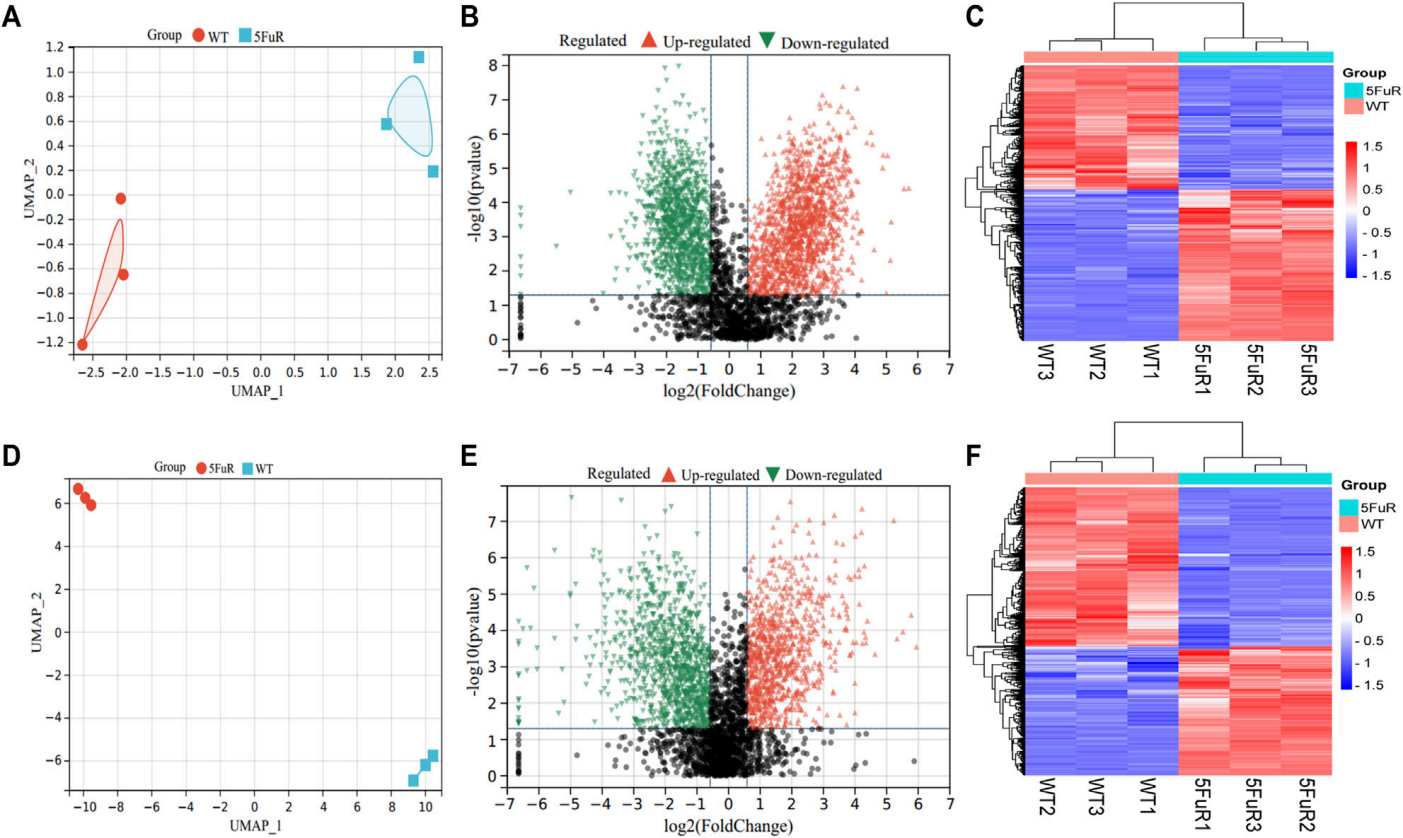
Differential expressed genes in 5-FU resistant CRC cells. **(A, D)** UMAP analysis of the proteomic data from parental and 5FU-resistant cells of HCT116 **(A)** and Lovo **(D)**. **(B, E)** Volcano plots showing the proteins with a statistically significant decreasing and increasing abundance in HCT116-5FuR **(B)** and Lovo-5FuR **(E)**. **(C, F)** The heatmap indicates the proteins that are expressed differentially between the parental and 5FU-resistant cells of the HCT116 cell line **(C)** and Lovo **(F)** cells.

To illustrate the mechanism of 5-FU resistance in CRC, the HCT116-5FuR and Lovo-5FuR and their corresponding parental cell lines underwent analysis using protein mass spectrometry. In the HCT116 and Lovo cell lines, a total number of 3683 and 3402 proteins were respectively discovered. Visualization of the abundance of proteins by UMAP (Methods) differentiated the proteome profiles, which clearly discriminated the proteomes of the WT and resistance (Figures 2A,D). In accordance with the outcomes obtained from protein mass spectrometry analysis, volcano plot and heatmap revealed 2175 proteins were significantly differentially expressed in HCT116-5FuR, in which 1195 underwent upregulation and 980 underwent downregulation (|fold change|>1.5, *p* < 0.05) (Figures 2B,C). Moreover, 813 highly expressed proteins and 998 downregulated proteins in Lovo-5FuR underwent screening (|fold change|>1.5, *p* < 0.05) (Figures 2E,F).

### 3.3 Resistant cells display increased glycolysis

**FIGURE 3:**
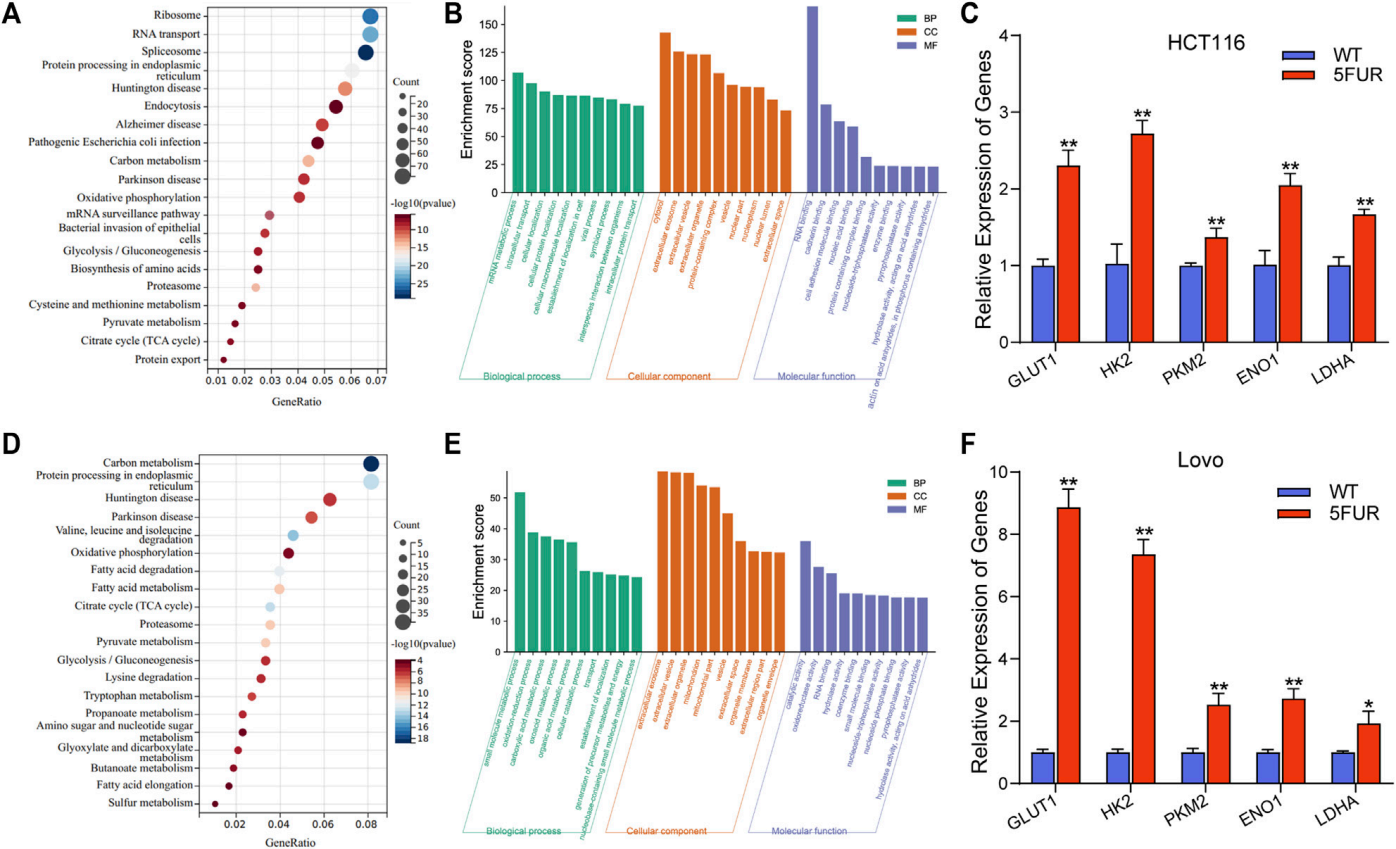
Resistant cells display increased glycolysis. **(A)** KEGG showing the significant enrichment of the top 20 pathways in HCT116-5FuR. **(B)** GO analysis of differentially expressed terms in parental and 5FU-resistant HCT116. **(D)** KEGG showing the significant enrichment of the top 20 pathways in Lovo-5FuR. **(E)** GO analysis of differentially expressed terms in parental and 5FU-resistant Lovo. **(C, F)** The mRNA expression of glycolytic enzymes HK2, PKM2, GLUT1, LDHA and ENO1 in parental and 5FU-resistant cells of HCT116 **(C)** and Lovo **(F)**. **p < 0.01, *p < 0.05.

The proteins that exhibit variable expression possess a range of molecular and biological functions, as discovered by the examination of KEGG and GO databases. The proteins in the HCT116-5FuR exhibited a notable enrichment primarily in metabolism, including Oxidative phosphorylation, Glycolysis/Gluconeogenesis and Citrate cycle (TCA cycle) (Figures 3A,B). Meanwhile, we found that the proteins in Lovo-5FuR were primarily enriched in Glycolysis/Gluconeogenesis (Figures 3D,E). Based on prior investigations, there exists a correlation between drug resistance and glycolysis. Consequently, our study focused on the examination of glycolysis in HCT116-5FuR and Lovo-5FuR cells. qPCR was conducted in order to ascertain Hexokinase 2 (HK2), Pyruvate kinase M2(PKM2), Glucose transporter type1 (GLUT1), lactate dehydrogenase A (LDHA), and α-Enolase (ENO1) expression level, which are recognized as pivotal enzymes involved in the glycolytic pathway. According to Figures 3C,F, the key enzymes were highly expressed in 5-FU resistant CRC cells, contrasted to the matched WT cells. The results of our study indicate that there was an observed elevation in glycolytic activity within colon cancer cells that had developed resistance to 5-FU treatment.

### 3.4 NOP58 is associated with CRC resistance

**FIGURE 4:**
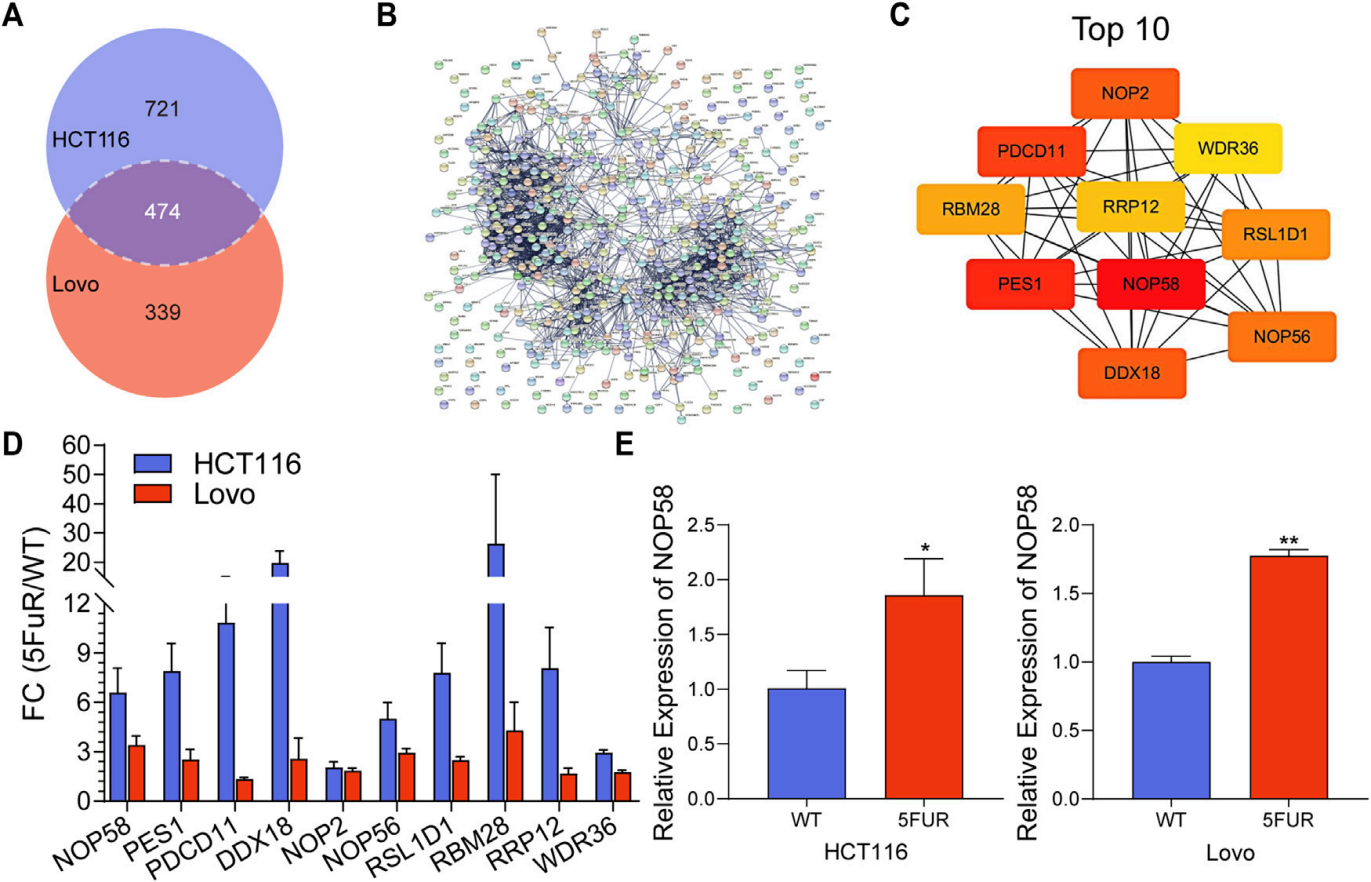
NOP58 is associated with CRC resistance. **(A)** The Venn diagram displayed the proteins that were found to be shared across the differentially expressed proteins in the HCT116-5FuR and Lovo-5FuR. **(B)** PPI network analysis of the overlapped proteins in **(A)**. **(C)** Top 10 overlapping protein hub genes in **(B)**. **(D)** The actually fold change expression of hub genes in HCT116-5FuR and Lovo-5FuR proteomic data. **(E)** qPCR showing the relative expression of NOP58 in parental and 5FU-resistant cells of HCT116 and Lovo. *p < 0.05.

In addition, 474 proteins overlapped between the upregulated proteins in HCT116-5FuR and Lovo-5FuR (Figure 4A). To further investigate the probable pathways, a study of protein-protein interactions (PPI) network was conducted on the subset of 474 proteins using the STRING database (Figure 4B). In addition, a comprehensive analysis was conducted to identify the top 10 hub genes, among which NOP58 emerged as the highest ranking gene (Figure 4C). We integrated the expression abundance of the top 10 hub genes in proteome of HCT116-5FuR and Lovo-5FuR (Figure 4D). In order to investigate the potential implications of NOP58 in the progression of drug resistance in colon cancer cells, we conducted an analysis to determine the expression levels of NOP58 in both HCT116-5FuR and Lovo-5FuR cell lines. According to Figure 4E, the mRNA levels of NOP58 were increased in HCT116-5FuR cells compared with HCT116 cells and the Lovo-5FuR cells demonstrated a notable elevation in mRNA levels of NOP58 in comparison to the Lovo cells. Taken together, NOP58 plays a vital role in CRC resistance.

### 3.5 NOP58 is correlated with CRC development

**FIGURE 5:**
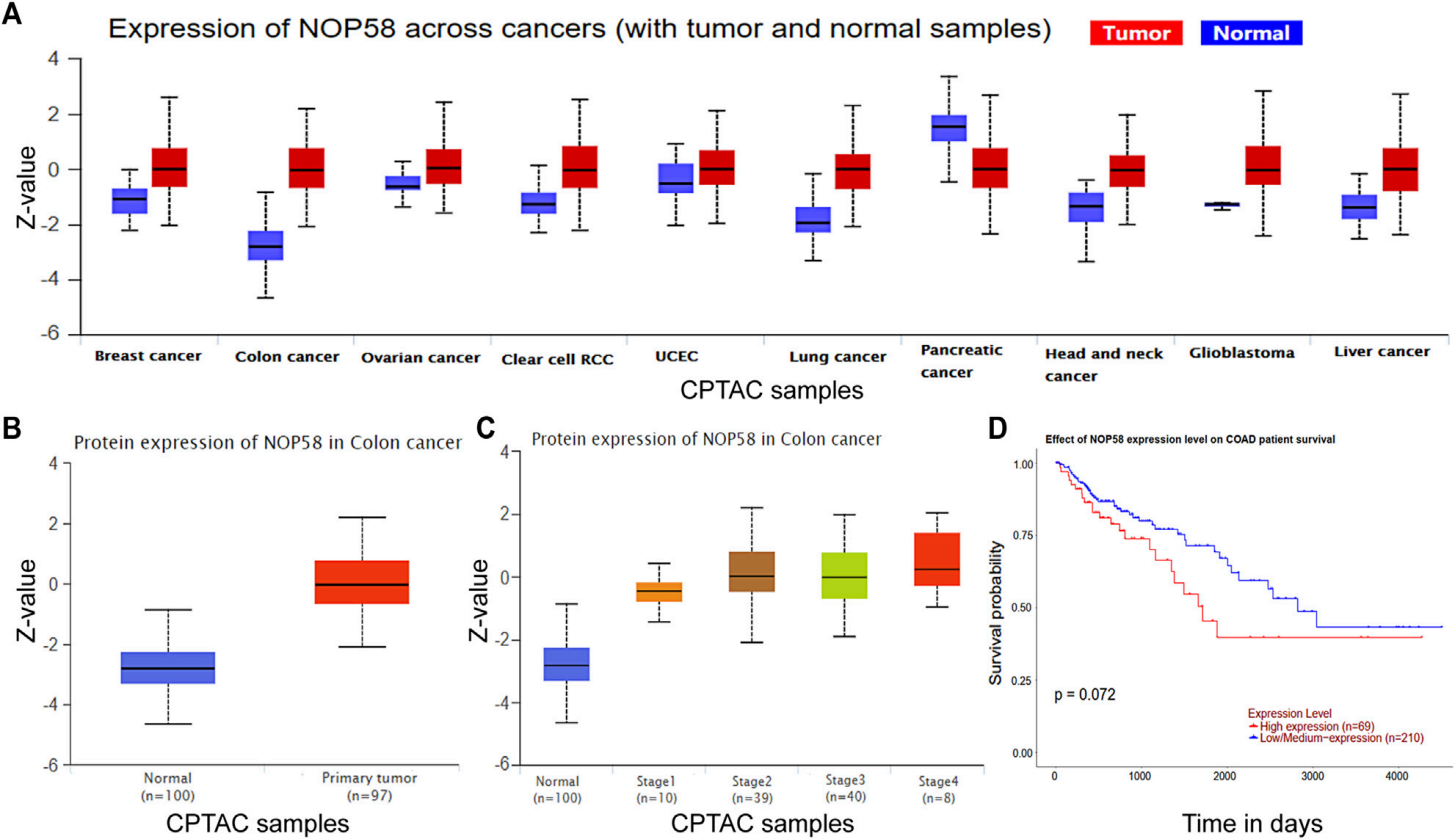
NOP58 is correlated with CRC development. **(A)** The CPTAC dataset showing the expression of NOP58 across cancers. **(B)** NOP58 protein expression in colon cancer. **(C)** NOP58 protein expression in different stages of colon cancer. **(D)** Kaplan–Meier survival curves of patients with COAD according to NOP58 expression.

We studied the NOP58 expression on series of cancers by the Clinical Proteomic Tumor Analysis Consortium (CPTAC) database (https://proteomics.cancer.gov/programs/cptac) in the UALCAN web, which revealed that NOP58 underwent overexpression across colon cancer, clear cell renal cell carcinoma (RCC), breast cancer, lung cancer, glioblastoma and liver cancer, head and neck cancer (Figure 5A). Furthermore, according to the CPTAC database, an elevation in the expression of NOP58 protein has been identified in CRC tissues in comparison to normal colorectal tissues (Figures 5B, C). Additionally, a strong positive association was seen between elevated levels of NOP58 and decreased overall survival rates among patients diagnosed with CRC (Figure 5D). Overall, NOP58 promoted the progression of colorectal cancer.

### 3.6 NOP58 knockdown overcomes the resistance to 5-FU by regulating glycolysis

**FIGURE 6:**
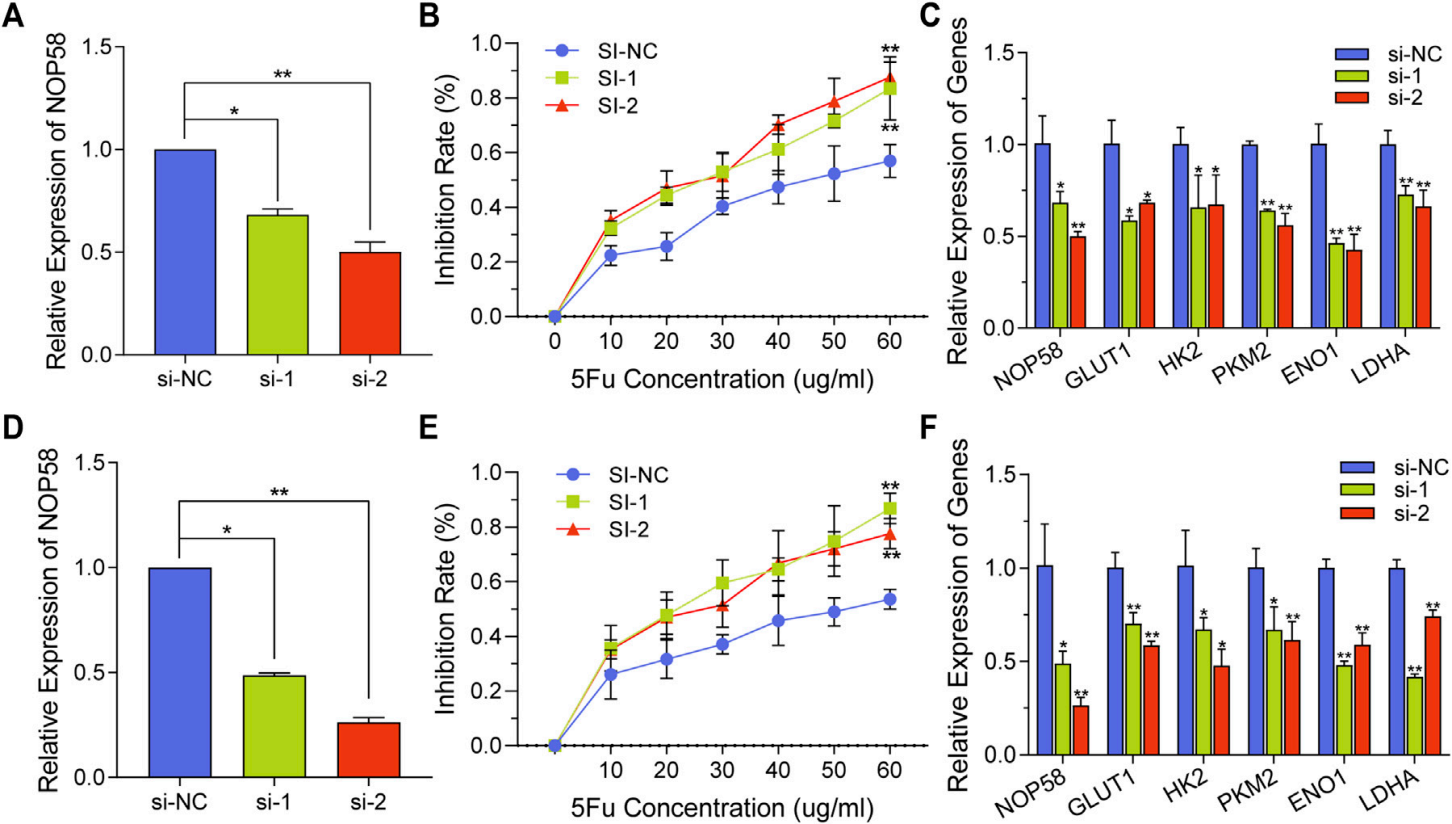
NOP58 knockdown overcomes the resistance to 5-FU by regulating glycolysis. **(A, D)** qPCR analysis demonstrating NOP58 expression level within HCT116-5FuR **(A)** and Lovo-5FuR **(D)** after the NOP58 siRNA or negative control siRNA (NC) were transfected. **(B, E)** The IC50 extents of HCT116-5FuR **(B)** and Lovo-5FuR **(E)** cells response to 5-Fu with or without NOP58 suppression. **(C, F)** The mRNA expression of glycolytic enzymes HK2, PKM2, GLUT1, LDHA and ENO1 in HCT116-5FuR **(C)** and Lovo-5FuR **(F)** transfected with negative control siRNA or NOP58 siRNA. **p < 0.01, *p < 0.05.

To investigate the impact of NOP58 on drug resistance, si-NOP58 or si-Con was transfected into HCT116-5FuR and Lovo-5FuR cells. Following a 48 h incubation period, qPCR was conducted, revealing that the introduction of si-NOP58 successfully suppressed the expression of NOP58 in both HCT116-5FuR and Lovo-5FuR cell lines (Figures 6A,D). HCT116-5FuR and Lovo-5FuR cells with or without transfections were subjected to incubation with varying amounts of 5-FU (10, 20, 30, 40, 50, 60 μg/mL) for 48 h. The IC50 value for 5-FU has been reduced in HCT116-5FuR and Lovo-5FuR underwent transfection with si-NOP58 in comparison to that in cells transfected via si-Con (Figures 6B,E). Furthermore, we conducted an assessment of the impact of si-NOP58 on the glycolytic pathway in CRC cells that had developed resistance to 5-FU. According to Figures 6C,F, si-NOP58 treatment caused decreased expression level of HK2, GLUT1, PKM2, ENO1, and LDHA. The findings of the study demonstrated that the inhibition of NOP58 expression resulted in a suppression of glycolysis in both HCT116-5FuR and Lovo-5FuR cells. Additionally, this silencing of NOP58 expression resulted in an elevated sensitivity to 5-FU.

## 4 Discussion

For almost 6 decades, 5-FU has served as the primary medication for single-drug and multi-drug chemotherapy, demonstrating its efficacy as a systemic treatment for the management of advanced and metastatic CRC (Correale et al., 2004; Rosmarin et al., 2014). However, a specific number of patients still acquired 5-FU resistance (Del Rio et al., 2007). Hence, the identification of ways to overcome resistance to 5-FU poses significant hurdles in the realm of clinical practice. Multiple pathways of resistance to 5-FU have been documented, encompassing alterations across the proportion of drug influx or efflux (Mansoori et al., 2017), intratumor heterogeneity (Mansoori et al., 2017), epigenetic factors (Kreso et al., 2013), tumor microenvironment (Gatenby et al., 2010), and 5-FU metabolic enzymes (Longley et al., 2003). The current investigation aims to provide a comprehensive understanding of the fundamental mechanism behind 5-FU resistance in CRC by examining its role in promoting glycolytic metabolism.

NOP58 was previously reported to contribute to maturation, stability, and localization of snoRNAs (Liang et al., 2019). We found that NOP58 was significantly upregulated within 5-FU resistant CRC cells. In addition, the downregulation of NOP58 expression led to elevated sensitivity of CRC cells to 5-FU, and decreased the expression of glycolytic enzymes. The implementation of this procedure may have had an impact on the responsiveness of cells treated with 5-FU chemotherapy.

An essential component of metabolic reprogramming in cancers is the heightened reliance on glycolysis as a means of energy production (DeBerardinis and Chandel, 2016). Furthermore, the high rate of glycolytic flow not only serves as an energy source but also offers a diverse range of raw materials for biosynthetic processes (Montal et al., 2015). The phenomenon of increased aerobic glycolysis performs a significant function in the proliferation of tumors, as it confers cancer cells with advantageous growth capabilities and resistance to therapeutic interventions (Shukla et al., 2017). The enzymes that perform a regulatory function in glycolysis have also been linked to the promotion of drug resistance across tumors (Xu et al., 2005; Bhattacharya et al., 2016).

The phenomenon of drug resistance in CRC cells towards vincristine and oxaliplatin can be effectively addressed by the genetic manipulation of polypyrimidine tract binding protein 1 (PTBP1) by means of knockout techniques (Cheng et al., 2018), a key regulator of the glycolytic pathway. The enzyme HK2 plays a crucial role in initiating the primary step that regulates the rate of glucose metabolism. Furthermore, the compound 2-DG, which acts as an inhibitor of HK2, has demonstrated the ability to counteract drug resistance in several *in vitro* models (Shi et al., 2019). The suppression of MCT1 significantly enhanced the chemosensitivity of human osteosarcoma cells (Zhao et al., 2014). The findings suggests that the suppression of glycolysis by the targeting of key enzymes involved in the glycolytic process could serve as a promising treatment strategy for effectively overcoming medication resistance across various cases.

In general, several techniques centered around molecular targets were suggested in order to counteract 5-FU resistance in CRC and enhance the efficacy of 5-FU treatment. Nevertheless, these endeavors have not yielded favorable outcomes. We demonstrated that NOP58 induced chemoresistance through glycolysis pathway. The current investigation offers a novel therapeutic strategy for the precise management of CRC individuals suffering from resistance to 5-FU therapy.

## Data Availability

The datasets presented in this study can be found in online repositories. The names of the repository/repositories and accession number(s) can be found in the article/Supplementary Material.
