# Supplementary material for: NOP58 induction potentiates chemoresistance of colorectal cancer cells through aerobic glycolysis as evidenced by proteomics analysis
Source: Front Pharmacol. 2023 Dec 12;14:1295422. doi: 10.3389/fphar.2023.1295422 (PMC10750250; doi:10.3389/fphar.2023.1295422)
Supplement: Supplementary file 2 [file Table1.docx]

Table S1

| Si-NOP58-1 | Si-NOP58-2 |
| --- | --- |
| S:  GCAGAAGCAUUAGCAGCAUTT | S:  CCAUAGUCCUGUUGUUAAUTT |
| AS:  AUGCUGCUAAUGCUUCUGCTT | AS:  AUUAACAACAGGACUAUGGTT |
