# Supplementary material for: NOP58 induction potentiates chemoresistance of colorectal cancer cells through aerobic glycolysis as evidenced by proteomics analysis
Source: Front Pharmacol. 2023 Dec 12;14:1295422. doi: 10.3389/fphar.2023.1295422 (PMC10750250; doi:10.3389/fphar.2023.1295422)
Supplement: Supplementary file 6 [file Table2.docx]

Table S2

| Gene | Forward: | Reverse: |
| --- | --- | --- |
| NOP58 | AAGCATGCAGCTTCTACCGT | GGTTTTGGCTGCCAGCATTC |
| Glut1 | TGGCATCAACGCTGTCTTCT | CTAGCGCGATGGTCATGAGT |
| HK2 | CTCCAAATCAGCCTCGGGAC | CCAAAGCACACGGAAGTTGG |
| PKM2 | TTCACTGAACGCAAACGGTG | CCCCACTCCCAGCATTACAG |
| LDHA | TTCAGCCCGATTCCGTTACC | CCGTAAAGACCCTCTCAACCA |
| ENO1 | CCTTAGAACCCACAGCCCAT | TGGTGCACTGCTTCCATCAA |
